# Characteristics of early pleural effusions after orthotopic heart transplantation: comparison with coronary artery bypass graft surgery

**DOI:** 10.1515/pp-2021-0143

**Published:** 2021-12-13

**Authors:** Anant Jain, Anusha Devarajan, Hussein Assallum, Ramin Malekan, Gregg M. Lanier, Oleg Epelbaum

**Affiliations:** Pulmonary Critical Care, Westchester County Medical Center, Valhalla, NY, USA; Internal Medicine, Westchester Medical Center, Valhalla, NY, USA; Cardiothoracic Surgery, Westchester County Medical Center, Valhalla, NY, USA; Transplant Cardiology, Westchester County Medical Center, Valhalla, NY, USA

**Keywords:** coronary artery bypass grafting, heart transplantation, pleural effusion

## Abstract

**Objectives:**

Pleural effusions appearing within the first 30 postoperative days following coronary artery bypass grafting (CABG) are classified as early and believed to be directly related to the surgery. The characteristics of such effusions are well-described. Orthotopic heart transplantation is also known to be complicated by pleural effusions; however, their characteristics have not been systematically reported. We assessed the features of early postoperative pleural effusions after heart transplantation and compared them to those of early effusions following CABG.

**Methods:**

We retrospectively collected demographic, clinical, and laboratory data for patients who underwent either orthotopic heart transplantation (study group) or CABG (comparison group) at our institution and whose postoperative course within 30 days was complicated by new or worsening pleural effusion that prompted drainage. Patients subjected to analysis consisted only of those with sufficiently complete laboratory profiles to permit adequate characterization of the nature of their pleural fluid.

**Results:**

Out of 251 orthotopic heart transplant recipients, seven (2.8%) were found to have sufficiently complete pleural fluid results to be included in the study group. Out of 1,506 patients who underwent CABG, 32 (2.1%) had sufficiently complete pleural fluid results and formed the comparison group. The radiological appearance of pleural effusions in both groups was similar: bilateral in at least half and exclusively moderate to large. Effusions complicating both surgeries were exudative in close to 90% of cases. For those with available leukocyte differential counts, the pleural fluid of the post-orthotopic heart transplantation group was more often neutrophilic (3/5, 60%), whereas the fluid of the post-coronary artery bypass grafting group was more often lymphocytic (22/32, 69%) and tended to be hemorrhagic (median RBC count 33,000 cells/µL vs. 10,000 cells/µL). None of the comparisons of pleural fluid characteristics between the two groups reached statistical significance.

**Conclusions:**

This small, descriptive study is the first to systematically report the fluid characteristics of pleural effusions complicating orthotopic heart transplantation within the first 30 postoperative days and to compare this group to those who developed effusions after CABG. Our findings revealed both similarities and differences in the pleural fluid characteristics between these two types of patients.

## Introduction

Coronary artery bypass grafting (CABG) is a common operation in the United States with an estimated 400,000 procedures performed annually [1]. Post-CABG pleural effusion is a well-characterized entity that complicates about one-half to two-thirds of these procedures, but large [2] or symptomatic [3] effusions occur much less frequently. Incising the pleura during harvesting of the internal mammary artery (IMA) is thought to increase the incidence of post-CABG pleural effusion, but pleural incision is not a consistent risk factor, implying a multifactorial process [4]. It is not surprising, therefore, that pleural effusions are also known to complicate cardiac surgery that does not involve traversing the pleural space. Orthotopic heart transplantation (OHT) is a comparatively rarer operation restricted to a small number of specialized centers. Although post-OHT pleural effusions have been characterized in general terms by prior investigators [5, 6], very little data exist about the pleural fluid (PF) characteristics of these effusions. Of particular interest are early effusions detected during the first 30 postoperative days and hence more likely to be related to the antecedent operation than to medical complications of OHT. Whether these early effusions after OHT are similar in nature to the better described early post-CABG effusions is unknown. The present study compares the PF characteristics of post-OHT patients who underwent sampling of a new or worsening pleural effusion within 30 days after surgery to those of post-CABG patients meeting the same criteria.

## Materials and methods

### Patients and methods

This single-center retrospective study was performed at Westchester Medical Center (WMC) located in Valhalla, New York. The medical records of all patients older than 18 years of age who received OHT at WMC between January 1, 2010 and May 31, 2020 were screened to identify those who underwent a pleural intervention (i.e., therapeutic thoracentesis, tube thoracostomy, thoracoscopy) within 30 days of transplantation, thus defining a group with early post-operative pleural effusion of sufficient clinical significance to merit a drainage procedure. Within this group, we identified a subset of patients who underwent PF testing. Cases fulfilling these criteria were potentially eligible for inclusion. Patients were excluded if missing laboratory results precluded definitive categorization of their effusion as transudative or exudative according to Light’s criteria [7] or according to the serum to PF total protein gradient, with a threshold value of <3.1 g/dL used to define an exudate [8]. It was decided *a priori* that patients would be excluded if available thoracic imaging prior to OHT revealed an ipsilateral pleural effusion that was of equal size or larger than the sampled post-operative effusion. Those remaining after the application of these inclusion and exclusion criteria constituted the OHT study population. To form the comparison group, we screened patients who underwent CABG between January 1, 2014 and December 31, 2019 and applied the same inclusion and exclusion criteria (i.e., new or worsening pleural effusion within the first 30 postoperative days that underwent sampling and adequate laboratory analysis). Relevant demographic, clinical, laboratory, and radiological information was extracted from the medical record for both groups. The primary objective of the study was to describe the PF characteristics of early post-OHT effusions and compare them to those of early post-CABG effusions.

The pleural effusions included in this study were divided according to size into two categories: small (i.e., occupying <1/3 of a hemithorax on the frontal chest radiograph closest to time of intervention) and moderate to large (i.e., occupying >1/3 of a hemithorax on the frontal chest radiograph closest to time of intervention). Effusion size was adjudicated by an experienced pulmonary physician (OE). In case of bilateral effusions, all reported characteristics were based on the sampled side.

### Statistical analysis

Values are presented as frequency and percentage for categorical variables and mean ± SD or median [IQR] for continuous variables unless otherwise specified. Fisher’s exact test (two-tailed) was used to compare categorical variables. Medians were assessed for statistically significant difference using the Mann–Whitney U test as long as both samples contained at least five entries. A p-value of <0.05 was considered to be statistically significant.

This study was approved by the Institutional Review Board of New York Medical College (protocol #12882). The requirement for informed consent was waived due to the retrospective nature of the study.

## Results

A total of 251 patients who received OHT between January 1, 2010 and May 31, 2020 were screened for the presence of new or increased post-operative pleural effusion within 30 days of surgery. In this population, 96 patients (38%) developed a new or worsening pleural effusion on imaging in that time period. Twelve patients (4.8% of the total and 13% of those with effusion) underwent PF sampling, but five were excluded due to incomplete PF studies, leaving seven patients eligible for analysis as the study group. The patient and effusion characteristics of this group are presented in Table 1. The indication for OHT was non-ischemic cardiomyopathy in five and ischemic cardiomyopathy in two. None of the patients in this group was bridged to OHT with a left ventricular assist device or extracorporeal life support. The immunosuppressive regimen consisted of tacrolimus, mycophenolate mofetil, and prednisone. One patient required treatment for refractory acute antibody-mediated rejection (AMR 1) with antithymocyte globulin, high-dose corticosteroids, rituximab, and plasma exchange. Between January 1, 2014 and December 31, 2019, a total of 1,506 patients underwent CABG, of whom 39 (2.6%) had information about PF drainage and sampling within 30 days of surgery. Seven were excluded for incomplete laboratory values; the remaining 32 formed the comparison CABG group. The characteristics of the CABG group are listed alongside the OHT group in Table 1. At least half of the patients in both groups had bilateral effusions, and the sampled effusion was moderate to large in all. No otherwise eligible patients were excluded from either group based on the presence of an ipsilateral preoperative effusion of equal or larger size than the postoperative one. Two of the seven OHT patients (29%) and 12 of the 32 CABG patients (38%) had a smaller preoperative effusion on the sampled side. The remainder had no detectable preoperative effusion. For the majority of OHT patients (5/7, 71%), chest radiography was the initial imaging modality that demonstrated the sampled effusion; for the majority of CABG patients (18/32, 56%), effusion was first detected by thoracic ultrasonography. All but two patients, both in the CABG group, underwent tube thoracostomy rather than thoracentesis.

**Table 1:** Comparison of patient and pleural effusion characteristics between the two groups.

Characteristic	Post-OHT (n=7)	Post-CABG (n=32)
Age, years	54 ± 12	69 ± 12
Gender		
Male	6 (85)	26 (81)
Female	1 (15)	6 (19)
Effusion laterality, n (%)		
Right	0	4 (13)
Left	3 (43)	12 (38)
Bilateral	4 (57)	16 (50)
Effusion size, n (%)		
Small	0	0
Moderate to large	7 (100)	32 (100)
Pre-operative effusion, n (%)		
Yes	2 (29)	12 (38)
No	5 (71)	20 (63)
Diagnostic modality, n (%)		
Chest radiography	5 (71)	5 (16)
Computed tomography	1 (14)	9 (28)
Ultrasound	1 (14)	18 (56)
Intervention, n (%)		
Thoracentesis	0	2 (6)
Tube thoracostomy	7 (100)	30 (94)

All values are expressed as mean ± SD or total (%). Percentages may not add up to 100 due to rounding. CABG, coronary artery bypass grafting; OHT, orthotopic heart transplantation.

The characteristics of PF in the OHT and CABG groups are summarized in Table 2. Six out of seven (86%) post-OHT effusions were exudative, which was similar to the percentage of exudative post-CABG effusions (28/32, 88%). Higher median red blood cell (RBC) count along with higher median PF lactate dehydrogenase (LDH) level were observed in post-CABG effusions compared to post-OHT effusions, a combination pointing to a bloodier nature of the former. Defined by an RBC count ≥100,000 cells/µL, two post-OHT effusions were grossly hemorrhagic (28%) compared to 10 (31%) post-CABG effusions, a non-significant difference. None of the post-OHT effusions qualified as a hemothorax (PF hematocrit >50% of serum hematocrit), nor were there any chylous or empyematous effusions in that group. There was one hemothorax and one empyema in the CABG group. The majority of post-OHT effusions were neutrophil-predominant (3/5, 60%), whereas the majority of post-CABG effusions were lymphocyte-predominant (22/32, 69%). There were no eosinophilic effusions (i.e., eosinophil percentage >10) encountered in either group.

**Table 2:** Comparison of the PF characteristics between the two groups.

Fluid parameter	Post-OHT patients (n=7)	Post-CABG patients (n=32)	p-Value
Total WBC, cells/µL	470 [1,295]	884 [1,482]	0.42
WBC type predominance			
Neutrophil	3 (60)^a^	10 (31)	0.32
Lymphocyte	2 (40)^a^	22 (69)	
Total RBC, cells/µL	10,000 [183,560]	33,000 [133,530]	0.37
Grossly hemorrhagic^b^	2 (28)	10 (31)	1.00
LDH, U/L	313 [6,198]^a^	371 [725]^c^	0.62
Total protein, g/dL	2.2 [0.5]	2.6 [1.1]	0.63
Glucose, mg/dL	87 [165]	153 [70]^d^	0.12
Triglycerides, mg/dL	30 [260]^a^	25 [23]^e^	0.17
Cholesterol, mg/dL	60 [39]^a^	32 [14]^f^	0.09
Exudate	6 (86)	28 (88)	1.00
Culture positive	0	1 (3)	NA
Cytology positive	0	0	NA

All values are expressed as median [IQR] or total (%). CABG, coronary artery bypass grafting; LDH, lactate dehydrogenase; NA, not applicable; OHT, orthotopic heart transplantation; RBC, red blood cell; WBC, white blood cell. ^a^Based on n=5. ^b^Defined as PF RBC count ≥100,000 cells/µL. ^c^Based on n=30. ^d^Based on n=24. ^e^Based on n=23. ^f^Based on n=23.

## Discussion

By comparing the PF characteristics of early post-OHT effusions with those of early post-CABG effusions, this small study adds to the scant existing literature on this underdescribed complication of OHT surgery. The typical early post-CABG effusion is a lymphocyte-predominant exudate [2], and this profile was also the most common in our CABG group. PF eosinophilia has been described as a prominent feature in one study [3] but it does not appear to be universal [2] even among bloody effusions and was not seen in our CABG patients, who had a lower median RBC count and rate of bloody PF than prior reports [2, 3]. The post-OHT effusions in our study were likewise exudative and free of eosinophils but were more often neutrophil-predominant than lymphocyte-predominant. The median RBC count in the post-CABG effusions was 3.3 times that of post-OHT effusions, leading to a higher median LDH level, though the percentage of grossly hemorrhagic PF was similar in the two groups. Chylous or infected fluid was not observed among the post-OHT effusions.

Pleural effusions occurring in the first 30 days following CABG are usually either clinically bland exudates with or without hemorrhage or a manifestation of postpericardiotomy syndrome (PPCS) as part of an inflammatory pleuropericarditis. The potent immunosuppressive regimen administered to OHT recipients postoperatively would be expected to eliminate the occurrence of PPCS, but in fact PPCS has been reported following OHT with the mechanism remaining speculative [9]. None of the patients in either group of our study was deemed to have pleural effusion in the setting of PPCS. Pleurotomy during LIMA harvesting [10] and ice cardioplegia [11] have both been associated with early post-CABG pleural effusion, but neither is part of the OHT operation. Both operations involve anticoagulation, so possible explanations for the seemingly bloodier nature of post-CABG effusions relative to post-OHT ones include pleural violation for LIMA harvesting or administration of antiplatelet therapy in the perioperative period. Lymphocyte predominance is a typical feature of leukocyte differential counts in post-CABG pleural effusions, so it was unsurprising that the majority of these were likewise lymphocytic in our study. In our small OHT group; however, we found more neutrophilic effusions than lymphocytic ones. This difference could reflect the impact of post-transplant lymphodepletional immunosuppression on the prevailing inflammatory cell response in the pleural space.

Our study differs from the few prior reports on post-OHT pleural effusions in both its presentation of specific PF characteristics and the inclusion of a post-CABG effusion comparison group. Our study is also distinguished by restriction to early pleural effusions occurring within the first 30 days postoperatively, a period during which effusions can more convincingly be traced to the recent cardiac surgery rather than to myriad other etiologies of pleural disease that could arise in immunosuppressed patients at a later stage. In our OHT population, the rate of clinically significant pleural effusions was 4.8%, a figure similar to the 6.7% incidence of effusions shown in the earliest published study on this subject that reported all pulmonary complications of OHT [12]. In that study, the vast majority of effusions occurred in the first six months. Analogously to our approach, these investigators defined a significant post-OHT pleural effusion as a compatible radiographic abnormality that prompted a management strategy. A more contemporary study of postoperative pulmonary complications of OHT, which, like our study, was limited to events within 30 days of surgery but defined pleural effusion strictly radiologically, showed an effusion rate of 26% [13] compared to 38% in our OHT group under these criteria. Misra et al. [5] have demonstrated that effusions deemed large based on occupancy of >25% of a hemithorax occurred in 17% of patients in the first year following OHT and were usually bilateral. In our study, all post-OHT effusions for which a drainage procedure was performed occupied at least 1/3 of the hemithorax on the intervention side, and in ≥50% of cases were indeed bilateral. The most granular description of post-OHT pleural effusions previously published belongs to a series of 50 patients reported by Ulubay et al. [6]. Nineteen of the 50 effusions were considered postoperative by the authors without further elaboration. A total of only 10 of the 50 patients underwent PF sampling, but it is not specified how many of these procedures were performed in the postoperative setting as defined by the investigators, and the actual PF laboratory values are not provided. Notably, three hemothoraces are reported in this ten-patient group. In contrast to our results, a significant minority of the 10 sampled patients (40%) had transudative effusions in this study.

We share the disappointment expressed by Misra et al. regarding the small number of PF aspirations found in their retrospective review: of the 12 patients with large effusions in their study, only four (33%) underwent thoracentesis. Though expressed differently, our rate of PF sampling was likewise underwhelming: 12 of 96 patients (13%) with new or enlarged pleural effusion within 30 days of OHT. Among those who underwent PF sampling, Misra et al. encountered inconsistent laboratory results, being able to confidently categorize only two of the four patients as having a transudative or exudative effusion. We faced a similar challenge in having to exclude five of the 12 patients (42%) who underwent pleural drainage because their incomplete laboratory profile negated definitive PF characterization.

Besides its small sample size in the OHT group that compromised statistical comparison with the CABG group, our study suffers from additional limitations, one of which is its retrospective nature. The decision to perform a pleural intervention was the product of provider discretion, so the patients who qualified for this study may represent a skewed population. Another limitation is the non-identical time periods for patient screening in the OHT vs. the CABG group. This was the result of the desire to extend the date range for OHT patients to maximize capture of study candidates, thereby compensating for the low number with pleural interventions and an even lower number with adequate laboratory profiles. Similarly, our allowance of a protein gradient <3.1 g/dL to serve as a criterion for ruling in an exudate permitted inclusion of patients lacking PF LDH results who thus did not qualify for full assessment by Light’s criteria. Finally, our method for adjudicating the size of an effusion and comparing postoperative size to preoperative size for effusions present before surgery contained an element of subjectivity.

## Conclusions

In this relatively small, descriptive study we provide the most detailed characterization of early post-OHT pleural effusions available to date and demonstrate that early post-OHT effusions are likely a different entity than the more familiar early post-CABG effusions. While both types of effusion are typically bilateral, exudative, and moderate to large when sampled, early post-OHT effusions tend to be more often neutrophilic rather than lymphocytic and less hemorrhagic compared to their post-CABG counterparts. Additional studies analyzing a greater number of patients with complete laboratory profiles would help support or refute these preliminary observations.
